# Microbe-Mediated Biosynthesis of Nanoparticles: Applications and Future Prospects

**DOI:** 10.3390/biom11060886

**Published:** 2021-06-15

**Authors:** Bhupendra Koul, Anil Kumar Poonia, Dhananjay Yadav, Jun-O Jin

**Affiliations:** 1 School of Bioengineering and Biosciences, Lovely Professional University, Phagwara 144411, Punjab, India; 2 Centre for Plant Biotechnology, CCSHAU, Hisar 125004, Haryana, India; anil_poonia2005@yahoo.com; 3 Department of Medical Biotechnology, Yeungnam University, Gyeongsan 38541, Korea; 4 Research Institute of Cell Culture, Yeungnam University, Gyeongsan 38541, Korea

**Keywords:** nanotechnology, nanoparticle, microbes, ecofriendly, nanomaterials bioremediation

## Abstract

Nanotechnology is the science of nano-sized particles/structures (~100 nm) having a high surface-to-volume ratio that can modulate the physical, chemical and biological properties of the chemical compositions. In last few decades, nanoscience has attracted the attention of the scientific community worldwide due to its potential uses in the pharmacy, medical diagnostics and disease treatment, energy, electronics, agriculture, chemical and space industries. The properties of nanoparticles (NPs) are size and shape dependent. These characteristic features of nanoparticles can be explored for various other applications such as computer transistors, chemical sensors, electrometers, memory schemes, reusable catalysts, biosensing, antimicrobial activity, nanocomposites, medical imaging, tumor detection and drug delivery. Therefore, synthesizing nanoparticles of desired size, structure, monodispersity and morphology is crucial for the aforementioned applications. Recent advancements in nanotechnology aim at the synthesis of nanoparticles/materials using reliable, innoxious and novel ecofriendly techniques. In contrast to the traditional methods, the biosynthesis of nanoparticles of a desired nature and structure using the microbial machinery is not only quicker and safer but more environmentally friendly. Various microbes, including bacteria, actinobacteria, fungi, yeast, microalgae and viruses, have recently been explored for the synthesis of metal, metal oxide and other important NPs through intracellular and extracellular processes. Some bacteria and microalgae possess specific potential to fabricate distinctive nanomaterials such as exopolysaccharides, nanocellulose, nanoplates and nanowires. Moreover, their ability to synthesize nanoparticles can be enhanced using genetic engineering approaches. Thus, the use of microorganisms for synthesis of nanoparticles is unique and has a promising future. The present review provides explicit information on different strategies for the synthesis of nanoparticles using microbial cells; their applications in bioremediation, agriculture, medicine and diagnostics; and their future prospects.

## 1. Introduction

Nanotechnology is an emerging branch of science which deals to with excising the structure of matter between 1 and 100 nanometers on the atomic, molecular and supramolecular level for the development of desired properties and functions and for diverse applications. In the last few decades, nanoscience has attracted the attention of the scientific community worldwide for the sustainable production of various nanoparticles (NPs), using innovative techniques, which finds applications in the pharmacy, medical diagnostics and disease treatment, energy, electronics, agriculture, chemical and space industries [1,2,3,4,5]. The novel applications of metal NPsin diverse industrial domains have also attracted the interest of researchers towards the synthesis and production of important metal NPs using simpler and efficient techniques [1,2,5]. It is expected that by 2030, the global nanomaterials market will grow by 20% [6]. The synthesis of NPs of a desired size, structure, monodispersity and morphology is crucial in terms of their various applications in nanoscience and associated nanobusiness [7].

**Figure 1:**
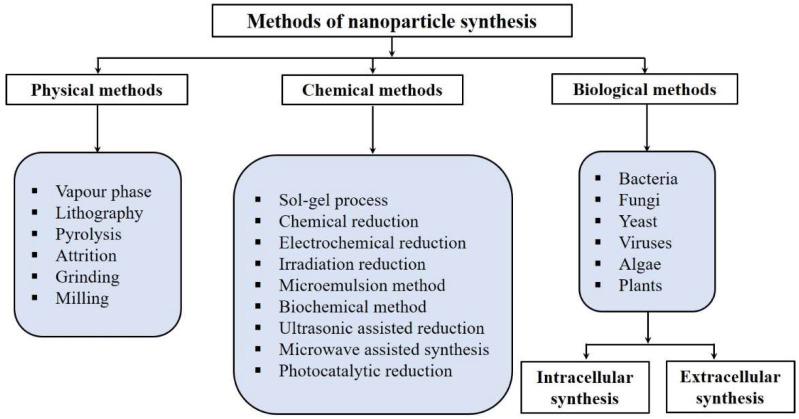
Different methods of NPs synthesis.

A recent advancement in nanotechnology is based on the synthesis of NPs/materials using novel techniques. NPs can be fabricated using physical, chemical and biological methods (Figure 1). The major physical methods used for the synthesis of nanoparticles include pyrolysis, physical vapor deposition (PVD), lithography, crushing, grinding, milling and ball processing. On the other hand, chemosynthesis of nanoparticles consists of sol gel synthesis, chemical reduction, electrolysis, CVD (chemical vapor deposition), photocatalytic reduction and microwave-assisted synthesis. 

The physical methods are inferior for the large-scale production of nanomaterials because of the lower yield of NPs, higher energy needs and higher input cost [8]. The chemical methods of NPs synthesis have been the methods of choice over the past few years due to the consumption of less energy during the reduction of metals and production of homogenous NPs with high accuracy [9]. Thus, these traditional methods of NPs synthesis are laborious, time consuming, hazardous and based on the use of toxic chemicals, which are unsafe (cytotoxic, genotoxic, carcinogenic) and act as potent environmental pollutants [10,11,12]. Moreover, the biomedical applications of NPs produced from chemosynthesis have been limited due to their instability and toxic nature [13,14].

Therefore, it is necessary to develop efficient, reliable, innoxious and ecofriendly techniques for the synthesis of NPs. This can be achieved by synthesizing NPs using natural sources, such as the biological system, microorganisms and their enzymes and biodegradable polymers.

**Figure 2:**
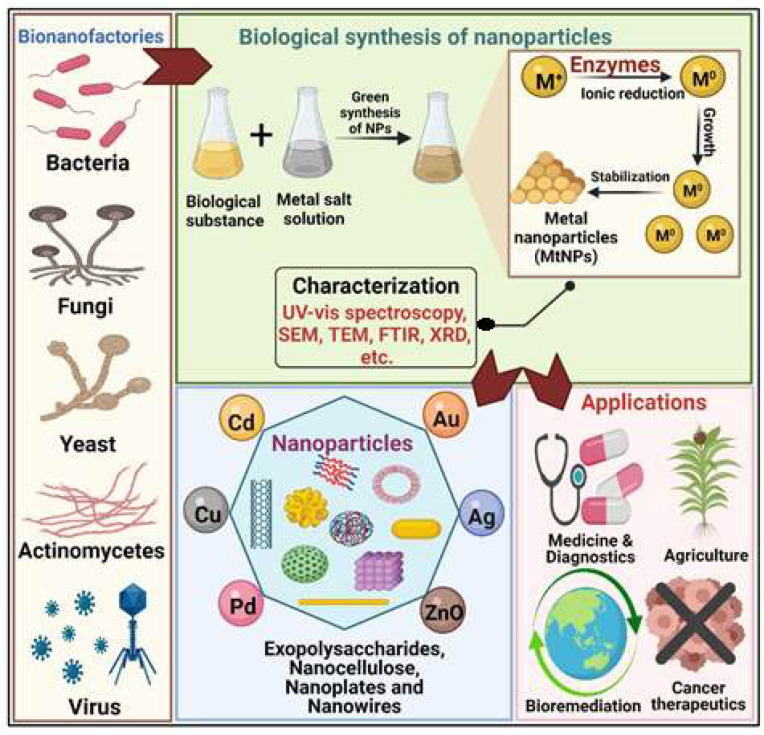
Depicts a schematic representation of the microbe-based biological synthesis of various nanoparticles, their characterization and applications.

NP synthesis using biological systems is rapid, feasible and ecofriendly. Moreover, the toxicity and size characteristics of the NPs can be controlled. Synthesis of NPs using bacteria, actinobacteria, fungi, yeast, microalgae and viruses has been explored by many researchers for the production of desired NPs [2,15,16,17,18,19,20,21]. Microbial cells have the natural ability to grow in diverse habitats and are fast growing and easy to maintain. Bacteria and microalgae possess the specific potential to fabricate distinctive nanomaterials, such as exopolysaccharides, nanocellulose and nanowires [22,23,24]. A schematic representation of microbe-based biological synthesis of different nanoparticles, its characterization and its applications is discussed in Figure 2.

Bacterial species like *Bacillus licheniformis*, *Pseudomonas deceptionensis*, *Pyrococcusfuriosus*, *Pyrobaculum islandicum*, *Pseudomonas aeruginosa,* etc., have been recently explored for the synthesis of silver (Ag), gold (Au), iron (Fe), nickel (Ni), zinc (Zn) and other important NPs [2,16,17,18,19,21,25]. It is interesting to note that the metabolic pathway of microbial machinery can also be altered through genetic engineering for the fabrication of NPs with specific physical, chemical and biological properties [26]. The application of microorganisms for the synthesis of NPs is unique but also has scope for improvement. The recent outbreak of COVID-19 has introduced a huge challenge to the field of medicine. Nanotechnology has the immense potential to strategically find solutions and to cope with such pandemic situations, as the NPs can be deployed for diagnosis and treatment of the COVID-19 disease [27]. Hence, the present review is focused on different strategies of the microbe-mediated synthesis of NPs; the applications of these NPs in bioremediation, pharmaceuticals, agriculture, medicine and diagnostics; and future prospects.

## 2. Strategies for Synthesis of Nanoparticles Using Microbes

The fabrication of NPs from living sources (bacteria, actinobacteria, yeast, fungi, algae and viruses) is quite safer than that of chemical and physical methods [2,15,16,17,18,19,20,21]. The fabrication of NPs using bacteria is preferred over other microbes as they can be cultured feasibly in artificial conditions with an ambient growth rate. Microbes can adapt in higher concentrations of metals and have the potential to reduce inorganic materials into NPs through their extracellular or intracellular routes [2,15]. Microbes absorb metal ions from their surrounding environment/media and convert these metallic ions into elemental form via an enzymatic reduction [28].

In the extracellular fabrication of NPs, microbes are grown in suitable media. The broth containing microbial cells is centrifuged, and the supernatant containing microbial enzymes is then used for the synthesis of NPs [29,30,31]. The supernatant containing reductase enzymes is allowed to react with the metal ions in a separate vessel. The bioreduction of metal ions in a cell-free supernatant results in the formation of NPs [32]. The characteristics of newly synthesized NPs (morphology, uniformity and interaction) are then determined by XRD (X-ray diffraction), SEM (scanning electron microscopy), TEM (transmission electron microscopy) and FTIR (Fourier transform infrared spectroscopy) (Figure 2) [33].

In the intracellular fabrication of NPs, the cellular mechanism of microbial cells is used for the synthesis of NPs. The microbial cultures are maintained in appropriate liquid media and the microbial biomass is washed with sterile distilled water followed by centrifugation to obtain the biomass pellet [14,16,34]. The microbial biomass is then allowed to react with an aqueous solution of metals. The solution containing the microbial biomass and metals is then cultivated at desired incubation conditions till a specific chromatic change is observed. The appearance of a specific color shows the formation of NPs. When a whitish yellow to yellow color appears, it suggests the synthesis of zinc and manganese NPs. While the appearance of a pale yellow to pinkish color suggests the synthesis of gold NPs, and a pale yellow to brownish color suggests the synthesis of AgNPs [35]. In this process, the metal ions (positively charged) are trapped within the cell wall (negatively charged) of microbial cells. The bioreduction of these metal ions within the cell wall by enzymes results in the formation of nanoclusters, and finally the NPs diffuse from the cell wall to the solution [32]. Hence, during the intracellular process the metal ions are absorbed by the microbial cells, and in the presence of cellular enzymes, metal ions are transformed into NPs within the cells, while, in the extracellular process, the metal ions are trapped outside the cell surface where they are reduced by cellular enzymes to form NPs [36].

### 2.1. Bacterial-Mediated Biosynthesis of Nanoparticles

Various metal NPs, such as Ag, Au, Cu, Se and Fe and metal oxide NPs, such as silver oxide (Ag_2_O) copper oxide (CuO), zinc oxide (ZnO), titanium oxide (TiO_2_), manganese oxide (MnO_2_), magnesium oxide (MgO), iron oxide (Fe_2_O_3_), etc., have been fabricated using bacterial cells as a bionanofactory [5]. These NPs can be used in diverse fields, including the production of third generation biosensors [37], biofilm development [38], biolabeling and cell imaging [39], sensoristic devices [40] and diagnostics [41]. Moreover, biofabricated NPs showed promising applications as anticancer agents [31,42,43,44,45,46,47,48], antimicrobials [31,42,43,49,50,51,52], antioxidants [44], anticoagulants [46] and antimigration and antiproliferative agents [45]. 

#### 2.1.1. Metal Nanoparticles 

##### Silver Nanoparticles (AgNPs)

It has been observed that the synthesis of AgNPs has increased immensely due to its wide range of applications in different fields [53]. As already mentioned, bacteria have an immense potential to withstand abiotic stresses and have the specific ability to reduce heavy metals into NPs. For instance, some of the bacteria, such as *Pseudomonas stutzeri* (facultative aerobe) and *P. aeruginosa* (facultatively anaerobe), can grow and survive at high concentrations of metals, including heavy metals [54,55]. 

The most commonly explored bacterial species for the synthesis of AgNPs include *Escherichia coli*, *Lactobacillus* sp., *Bacillus cereus*, *Acinetobacter* sp., *Pseudomonas* sp., *Corynebacterium* sp. and *Klebsiella pneumonia* [32,56,57]. Klaus-Joerger et al., 1999 were the first to report the fabrication of AgNPs (size less than 200 nm) using the bacterial strain *Pseudomonas* *stutzeri* AG259 [58]. They observed that *Pseudomonas stutzeri* AG259 has the ability to accumulate Ag atoms in the cellular periplasmic space and can be used to synthesize different sizes of AgNPs (larger size, up to 200 nm) when placed in a concentrated solution of AgNO_3_ (50 mM). The difference in size of NPs was due to the incubation of bacterial cells to different metal conditions. Such types of bacterial strains can be used for the industrial recovery of Ag. However, the exact process of the fabrication of AgNPs by this bacterial strain is still not clear. Nair and Pradeep (2002) demonstrated that a common bacterial strain of *Lactobacillus* found in buttermilk could be deployed for the fabrication of Au and AgNPs [59].

Shahverdi et al., 2007 reported the quick production of AgNPs by reduction of aqueous Ag ions using supernatants from cultures/cell-filtrate of *Escherichia* *coli*, *Klebsiella pneumoniae* and *Enterobacter cloacae* [60]. They observed that cell-filtrate rapidly reacted with Ag ions, and AgNPs were produced within 5 min of reaction. However, among the different bacteria used, the supernatant from *Enterobacter cloacae* (*Enterobacteriaceae*) was found to be most potent for the speedy fabrication of AgNPs [61]. Kalimuthu et al., 2008 studied the formation of AgNPs using *B. licheniformis* and observed that the biomass of *B. licheniformis* reduced the Ag ions (aqueous) into AgNPs [62]. The enzyme nitrate reductase led to the biosynthesis of stable NPs within 24 h of reaction. Hence, to enhance the speed of the process, it is very important to use suitable microbe(s).

In another study, Parikh et al., 2008 studied the molecular mechanism of biofabrication of AgNPs in *Morganella* sp. RP-42 extracted from insect midgut. They identified three homologous genes (*silE*, *silP*, and *silS*) in this bacterium [63]. They further illustrated that the *silE* gene isolated from *Morganella* sp. has 99% sequence homology with the previously discovered *silE* gene which encodes a periplasmic silver-binding protein. *Morganella* sp. RP-42 was successfully used for the fabrication of extracellular crystalline AgNPs (size 20 nm). This is a unique report which suggests the molecular evidence of Ag resistance in bacterium and can be deployed in the fabrication of NPs [63]. In another study, Mokhtari et al., 2009 studied the effect of variable visible-light irradiation on the synthesis of AgNPs by using the supernatant obtained from cultures of *Klebsiella* *pneumoniae* and aqueous AgNO_3_ [64]. They fabricated AgNPs of a consistent size (average size of 3 nm) and shape. Similarly, Nanda and Saravanan (2009) obtained AgNPs by extracellular reduction of aqueous Ag ions in bright conditions for 5 min, using the supernatant obtained from cultures of *Staphylococcus aureus* [65].

Ramanathan et al., 2010 studied the effect of temperature on the biosynthesis of AgNPs using the bacterium *Morganella psychrotolerans*. The authors standardized the different growth attributes and studied their effect on the morphology of newly synthesized AgNPs [66]. They observed that at a temperature of 25 °C, a combination of spherical nanoparticles and triangular and hexagonal nanoplates were formed, while at a temperature of 20 °C, spherical AgNPs (size 2–5 nm) were obtained. When the temperature was further decreased from 20 to 15 °C, it resulted in the formation of a combination of spherical NPs and nanoplates. When the temperature was further reduced to 4 °C, only a few larger sized spherical NPs (70–100 nm in size) were produced. This showed that the incubation temperature influences the size and symmetry of NPs. Hence, it is necessary to standardize reaction conditions for obtaining desired NPs.

In another study, Prakash et al., 2011 investigated the extracellular synthesis of spherical AgNPs (size 10–30 nm) using *Bacillus cereus*. The antibacterial effect of fabricated AgNPs was also studied against *E. coli* (Gram-ve) and *Streptococcus* (Gram+ve) using concentrations of AgNPs, and it was found that AgNP concentrations as low as 50 ppm could effectively check the bacterial growth [67]. Sunkar and Nachiyar (2012) studied the potential of the *Bacillus* species towards the synthesis of AgNPs and observed that the *Bacillus* species fabricated the AgNPs (size 10 to 20 nm) extracellularly and reduced the Ag ions in solution [68].

It has been observed that bacterial strains that thrive in various extreme habitats can be used for the biosynthesis of NPs. Moreover, different parameters also effect the biosynthesis of NPs from microbes. Lin et al., 2001 demonstrated that the formation of NPs via reduction involves many factors, including desired environmental attributes (pH and temperature) and availability of organic functional groups on the cell wall of bacterium which initiate/induce the reduction of metal ions [69]. Thus, the shape, symmetry, composition and size of NPs can be governed by the environmental conditions, such as culture medium, temperature, reactant concentrations, reaction time, pH and metallic salt to be reduced [59,70,71,72]. For instance, *Bacillus cereus* isolated from soil contaminated with heavy metal was successfully used for the extracellular biofabrication of AgNPs at standard temperature [29]. These biosynthesized AgNPs were reported to be employed in an array of applications as they exhibited good surface plasmon polariton properties. In another study, Kulkarni et al., 2015 observed that the radiation resistant strain of bacterium *Deinococcus radiodurans* reduces the AgCl solution and biofabricates extracellular AgNPs [73]. In a recent work, Yumei et al., 2017 observed the effect of temperature, pH and metal ion concentration on the biofabrication of AgNPs using *Arthrobacter* sp. They elucidated that at 7–8 pH and 70 °C, *Arthrobacter* sp. fabricated face centered cubic AgNPs (size 9–72 nm) at a lower concentration of AgNO_3_ (1 mM) [74], while at the same temperature and at a higher concentration of AgNO_3_ (3 mM), *Arthrobacter* sp. fabricated bunches of AgNPs. Moreover, it was observed that the AgNPs were only synthesized at pH 7–8, and at pH less than 5 and more than 8, NP synthesis ceased. Furthermore, it was reported that when the incubation temperature was increased from 70 to 90 °C, the fabrication time of NPs was also reduced from 10 to 2 min. This work demonstrated the direct influence of reaction conditions on the biosynthesis of NPs [74].

The use of genetically engineered microorganisms for the fabrication of homogenous NPs of a desired shape and size is a novel strategy. Recently, approaches such as the discovery of genes responsible for the fabrication of nanoparticles, the introduction of foreign gene and cellular reprogramming have been under investigation for the improvement of wild type strains of microorganisms for the biogeneration of NPs with desired characteristics [75]. For instance, Lin et al., 2014 investigated that the CusCFBA silver/copper efflux system present in *E. coli* strain (116AR) governs the expression of genes of membrane proteins. When this strain of *E. coli* (116AR) was exposed to AgNO_3_for a long time, it accumulated the AgNPs in its periplasmic space [76]. Similarly, Ramanathan et al., 2013 demonstrated the fabrication of AgNPs using a mutant strain of the *E. coli* (silver-resistant)-expressing CusCFBAAg/Cu system. The mutant strain accumulated the AgNPs in the periplasm [77]. Recently, Yuan et al., 2019 also reported the fabrication of AgNPs by using a transformed strain of *E. coli* containing metallothionein in the gene from *Candida albicans*. The authors concluded that the transformed *E. coli* can be further used for the enhanced production AgNPs [78].

Jin-Zhou et al., 2000 found that the functional groups that occur on the cell wall of dried biomass of the *Bacillus megaterium* D01 and *Lactobacillus* sp. A09 bacteria reduce the Ag ions and facilitate the fabrication of AgNPs [79]. Ahmad et al., 2003 biosynthesized AgNPs based on the process of NADH-dependent reductase enzyme, which releases electrons and oxidizes to NAD in the bacterium *Pseudomonas stutzeri* AG259 [80]. Cynobacteria have also been successfully used for the biosynthesis of AgNPs via enzymatic reduction. For example, Lengke et al., 2007 demonstrated the biosynthesis of AgNPs using a filamentous cynobacteria *Plectonema boryanum* UTEX 485. In this study, the cynobacteria were incubated with AgNO_3_ solutions for 28 days at 25–100 °C. The interaction between AgNO_3_ solutions and cynobacteria leads to the formation of up to 200-nm-sized octahedral Ag platelets and spherical AgNPs [81].

##### Gold Nanoparticles (AuNPs)

The bacterial cell machinery has also been utilized for the biofabrication of AuNPs by employing microbes [82]. Beveridge and Murray (1980), for the first time, demonstrated the intracellular fabrication of AuNPs by employing the bacteria *Bacillus subtilis* 168 and observed the existence of octahedral AuNPs (5–25 nm) [83]. As already mentioned, that medium conditions largely influence the synthesis of NPs. Konishi et al., 2004 studied the biosynthesis of AuNPs using the *Shewanella algae* and the effect of solution pH on the size and site of NP synthesis [84]. Mesophilic bacterium *Shewanella algae* were incubated in a medium at different pH levels. It was observed that the pH of solution affects the size and site of the NPs in *Shewanella algae.* The AuNPs of size 10–20 nm were formed in the periplasmic space of bacterium when the pH of the solution was 7.0, while 50–500- nm-sized AuNPs were accumulated extracellularly when the pH of the solution was dropped to 1. Similarly, He 2007 also studied the effect of pH on the size and shapes of AuNPs using the bacterium *Rhodopseudomonas capsulata*. They used an aqueous chloroauric acid (HAuCl_4_) solution with different pH (4–7) for the synthesis of AuNPs using the biomass of *R. capsulata*. They reported that spherical AuNPs (10–20 nm) were produced when the solution pH was 7.0, while Au nanoplates were formed when the pH of the solution was 4.0. Hence, the pH of the solution is a governing factor which controls the shape and site of the biosynthesis of AuNPs [22]. The same study described the effect of pH and metallic ion concentration on the morphology of biosynthesized AuNPs using *Rhodopseudomonas capsulata.* The authors reported that at lower concentrations of AuCl_4_ and at 6.0 pH, spherical AuNPs (size 10 to 20 nm) were synthesized, while at higher concentrations of AuCl_4_, gold nanowires were formed at a similar pH of 6.0. These findings are in accordance with the observations of Klaus et al., 1999, who reported that the size of NPs is affected by variations in incubation conditions [58]. Husseinyet al., 2007 demonstrated the extracellular synthesis of AuNPs using *Pseudomonas aeruginosa* enzymes [85]. However, in another study it has been shown that the bacterial enzymes do not participate in the synthesis of AuNPs [86]. For instance, Liu et al., 2009 reported the use of dried cells of *Bacillus megaterium* for the nonenzymatic synthesis of AuNPs [87]. Similarly, Sneha et al., 2010 also observed the nonenzymatic reduction process in the production of NPs in bacterium *Corynebacterium* sp. [86]. On the other hand, Correa-Llantén et al., 2013 demonstrated the enzymatic-reduction-mediated intracellular biosynthesis of quasihexagonal shaped and 5–50-nm-sized AuNPs using the bacterium *Geobacillus* sp. strain ID17 [88]. They concluded that during the metallic reduction, NADH acts as a cofactor, and cellular enzymes catalyze the biosynthesis process of AuNPs. In another study, Srinath et al., 2018 reported the biosynthesis of 20–25-nm-sized, well-dispersed AuNPs using the bacterium *Bacillus subtilis*. They concluded that these AuNPs could be efficiently catalyzed by the decomposition of methylene blue and could also be used for the bioremediation of other toxic dyes [89].

Different groups of cyanobacteria have also been used for the biosynthesis of AuNPs. Lengkeet al., 2006 and Brayneret al., 2007 have exploited *Plectonema* sp. for the fabrication of AuNPs [90,91]. Govindaraju et al., 2008 observed that *Spirulina platensis* (single-cell protein) fabricated AuNPs and Au core-Ag shell NPs [92].

Genetically engineered strains have also been successfully used for the biofabrication of AuNPs. For example, Attaran et al., 2016 demonstrated the biosynthesis of AuNPs using different genetically engineered strains of microorganisms. Recombinant strains of *P. Savastanoi*, *R. solanacearum*, *P. syringae* and *V. fischeri* produced 40-, 15-, 30- and 20-nm-sized AuNPs at a neutral pH. It was reported that the NADH-dependent reductase and NADH were responsible for the biosynthesis of AuNPs [93].

##### Other Important Metal Nanoparticles

Brock and Gustafson (1976) observed that when the bacteria *Sulfolobus acidocaldarius*, *Thiobacillus* *thiooxidans* and *Thiobacillus ferrooxidans* were grown on elemental sulfur containing energy sources, these bacteria can efficiently reduce the ferric ion to a ferrous state. *T*. *thiooxidans* also efficiently reduced the ferric iron aerobically at a low pH medium and produced stable ferrous iron [94]. The ferrous ions formed were also stable to auto-oxidation.

Some other reports are also available where the process of biomineralization results in the formation of NPs; for instance, *Escherichia coli* K12 synthesize Tellurium (Te) [95], stagnant cells of *Geobacter metallireducens* (strain GS-15) and *Shewanella putrefaciens* cause the enzymatic reduction of Tc (VII) [96], and *Rhodospirillum rubrum*, *Enterobacter cloacae* and *Desulfovibriode sulfuricans* reduce selenite to selenium (Se). It has also been reported that some of the bacteria can fabricate inorganic material NPs [97]. For example, Lovley et al., 1987 synthesized intracellular magnetite NPs using magnetotactic bacteria [97]. Mullen et al., 1989 observed that bacterial cells can efficiently bind to metallic cations and found that *P*. *aeruginosa*, *E*. *coli*, *B. subtilis* and *B. cereus* have a good ability to remove Cu^2+^, La^3+^, Cd^2+^ and Ag+ from aqueous solution [98].

Watson et al., 1999 reported the biosynthesis of magnetic iron sulfide (FeS) NPs on the surfaces of sulfate-reducing bacteria. These highly magnetic FeS nanoparticles of 20 nm in size were isolated from the solutions using a 1 Tesla high gradient field. The magnetic NPs were biofabricated due to bacterial iron reduction and acted as a physical indicator of geological setting via biological activities [99]. Biosynthesized FeS magnetic NPs can adsorb an array of heavy metals and few anions; hence, they can be applied as a potential adsorbent of heavy metals in bioremediation. It has been observed that the biofabrication of magnetite NPs is quite slow and requires complete anaerobic conditions. However, Bharde et al., 2005 synthesized NPs of magnetite using the nonmagnetotactic bacteria *Acinetobacter* spp. in complete aerobic conditions [100]. The magnetite nanoparticles were synthesized extracellularly in aerobic conditions when incubated with aqueous iron precursors, and these NPs exhibited good magnetic properties. In another study, the strain of *Lactobacillus* was efficiently deployed for the fabrication of titanium NPs [101].

Semiconductor NPs have also been fabricated using bacterial cellular machinery. Cunningham and Lundie (1993) reported that when *Clostridium thermoaceticum* cells were incubated in a medium containing cysteine hydrochloride (sulfide and CdCl_2_ source), it results in the formation of CdS NPs in the medium as well as on the surface of the bacterial cells [102]. Sweeney et al., 2004 also documented the intracellular fabrication of semiconductor cadmium sulfide (CdS) NPs using *E. coli.* They observed that the incubation of *E. coli* with sodium sulfide (Na_2_S) and cadmium chloride (CdCl_2_) results in the fabrication of cadmium sulfide (CdS) nanocrystals [103]. However, the synthesis of the nanocrystals was largely influenced by the physiological parameters, and the maximum yield (20-fold) of the nanocrystals was observed when the bacterial cells entered into the stationary phase.

A quick biosynthesis technique for the fabrication of spherical CuNPs (size 8–15 nm) was demonstrated by Varshney et al., 2011 using *Pseudomonas stutzeri* (nonpathogenic bacteria). In the same study, they also synthesized cubical copper NPs (size 50–150 nm) from electroplating wastewater, using the *P. stutzeri* strain isolated from soil [104]. It has also been reported in various studies that the biomineralization of metal ions results in the formation of NPs; for example, *Shewanella algae*, *Pantoea agglomerans*, *Lactobacillus acidophilus* and *Azoarcus* sp. reduce selenite to Se [34,44,105]. The nanocrystals of Fe_3_O_4_ and/or Fe_3_S_4_ are also known to be bacterial magnetosomes. Bacterial magnetosomes have been biofabricated using both nonmagnetotactic and magnetotactic bacteria via the biomineralization process [106]. These bacterial magnetosomes are of a narrow size, highly pure, have defined crystal morphology, are stable at an ambient temperature [107] and can be used for the production of chip-based biosensors for the determination of toxicity, for cancer treatment and in molecular imaging [108,109,110].

Various types of heavy metal nanoparticles (platinum (Pt), tellurium (Te), palladium (Pd), etc.) have also been synthesized for specific applications in various fields. For instance, Zonaro et al., 2017 demonstrated that the bacterium *Ochrobactrum* sp. can be used to convert toxic tellurite oxyanions into useful TeNPs [111]. In another recent study, electrochemically active biofilms of the bacterial strain *Shewanella loihica* PV-4 were used to synthesize Pt and PdNPs (size 2–7 nm) [112].

Similar to Ag and AuNPs, some other important NPs like CdS, Cu and magnetic NPs have also been synthesized using genetically engineered strains of bacteria. For instance, Kang et al., 2008 used a recombinant strain of *E. coli* (JM109) having altered gamma-glutamylcysteine synthetase and phytochelatin synthase genes (from *Schizosaccharomyces pombe*) for the production of cadmium sulfide (CdS) nanocrystals [26]. Similarly, Chen et al., 2009 also reported the fabrication of CdSNPs using two recombinant strains of *E. coli* (strain ABLE C) and observed that the rate of production of NPs using an engineered strain of *E. coli* was around 2.5 times better than the wild-type strain [113]. In another study, Jung et al., 2012 demonstrated the biofabrication of homogenous magnetic NPs using a recombinant *E. coli* strain expressing metallothionein and phytochelatin synthase genes [114]. Kolinko et al., 2014 studied the different genes (*mamAB*, *mamGFDC*, *mamXY*, and *mms*6) responsible for the biomineralization of magnetosome and the pathway of magnetite NP synthesis in the bacterium *Magnetospirillum gryphiswaldense*. The authors transferred the magnetosome biomineralization pathway (mam genes) into a heterologous host *Rhodospirillum rubrum* and fabricated magnetite NPs of 24 nm in size encapsulated by the protein shell [115]. In a recent study, Choi et al., 2018 demonstrated the biosynthesis of more than 60 different nanomaterials using genetically engineered *Escherichia coli* and concluded that the expression of the metal reductase gene in a recombinant strain of *Escherichia coli* could be employed for the biosynthesis of various chalcogenides, such as cadmium selenide, zinc sulfide and cadmium sulfide [116].

#### 2.1.2. Metal Oxide Nanoparticles

Apart from metal NPs, bacteria have also been used to biosynthesize metal oxide NPs, such as Ag_2_O, CuO, ZnO, TiO_2_, MnO_2_, MgO and Fe_2_O_3_, which have a wide range of applications in the food industry, biomedicine, biosensors, optoelectronics, sunscreens and antimicrobial agents. For instance, spherical and triangular Ag_2_ONPs (size 2–20 nm) were successfully synthesized using Ag-resistant *Lactobacillus mindensis* isolated from an X-ray fixer solution [117]. The green-synthesized Ag_2_ONPs have strong antimicrobial activity and can be used in wound healing and skin creams for treatment of bacterial infection [118]. Vithiya et al., 2014 also reported that spherical Ag_2_ONPs (size 10–40 nm) biofabricated using *Bacillus thuringiensis* SSV1 exhibited strong antimicrobial activity against both Gram-negative (*E. faecalis*, *E. coli*, *P. mirabilis*, *Pseudomonas* sp.) and Gram-positive (*S. aureus*) bacteria [119]. CuONPs are another class of important metaloxide NPs which have a wide range of biological properties [120]. In a unique study, Hassan et al., 2019 extracellularly biofabricated CuONPs from two isolates of actinobacteria *Streptomyces* sp. (isolates Oc-5 and Acv-11) extracted from the medicinal plant *Oxalis corniculata* L. The spherical 78–80-nm-sized CuONPs obtained in this study showed excellent antibacterial, antifungal and antioxidant activity [121]. In a recent report, CuONPs have been synthesized using a cell-free supernatant of the marine bacterium *Streptomyces* sp. MHM38 [122]. The antimicrobial activity of spherical CuONPs (size 1.72–13.49 nm) was tested against different bacteria (*E. coli* ATCC 8939, *E. faecalis* ATCC 29212, *P. aeruginosa* ATCC 9027, *S. typhimurium* ATCC 14028), fungi (*A. niger*, *F. solani*, *R. solani*) and yeast (*C. albicans* ATCC 10237). It was concluded that CuONPs showed maximum antimicrobial activity against *C. albicans* ATCC 10237. ZnONPs have been widely studied and green synthesized due to their vast applications in biomedicine, the food industry, agriculture and cosmetics. Recently, Mohd Yusof et al., 2020 deployed the supernatant and cell biomass of the zinc-tolerant bacteria *Lactobacillus plantarum* TA4 in order to synthesize ZnONPs. TEM analysis suggested the biosynthesis of irregularly shaped ZnONPs (size 191.8 nm) using the cell biomass and flower-like pattern ZnONPs (size 291.1 nm) using a cell-free supernatant of bacteria. The antimicrobial activity of biosynthesized ZnONPs was studied against Gram-positive (*S. epidermidis* and *S. aureus*) and Gram-negative (*Salmonella* sp. and *E. coli*), and it was observed that ZnONPs were more effective against Gram-positive bacteria [123]. Another amnestic work reports the biofabrication of ZnO, MnO_2_ and MgONPs by deploying a rhizophytic bacteria *Paenibacillus polymyxa* strain Sx3 [124]. TEM and SEM analysis revealed that the ZnONPs (size 56.1–110.0 nm), MgONPs (size 10.1–18.8 nm) and MnO_2_NPs (size 19.8–63.9 nm) were cubic, sheet-like and spherical in shape, respectively. All three metal oxide NPs exhibited significant antimicrobial activity against the bacterium *Xanthomonas oryzae* pv. *Oryzae* (*Xoo*) responsible for bacterial leaf blight in rice [124]. TiO_2_ is another important metal oxide NP which has high refractive index (*n* = 2.4), is highly stable and possesses specific magnetic, optical, electrical and thermal properties [125]. Due to these unique features, TiO_2_NPs finds applications in electronic devices, PV shell, sensing instruments, splitting and photocatalytic degradation [126,127]. An interesting study showed that biosynthesized TiO_2_NPs using *B. amyloliquefaciens* exhibited reasonable photocatalytic activity against the textile dye Reactive Red 31 (RR31) [128]. The spherical TiO_2_NPs (size 22.11–97.28 nm) were doped with metals, such as La, Zn, Pt and Ag, to increase the photocatalytic activity of biosynthesized TiO_2_NPs. It was observed that TiO_2_NPs doped with Pt exhibited higher photocatalytic activity (90.98%) against RR31 dye than the undoped ones (75.83%). In a unique report, a consortium of three bacteria (*Micrococcus aloeverae*, *Micrococcus lylae*, *Cellulosimicrobium* sp.) isolated from the rhizosphere of sorghum plant along with root extract of sorghum was used to synthesize TiO_2_NPs [129]. The enzyme glucosidase present in bacteria acted as bioreducing agent. TEM and FTIR analysis confirmed the biosynthesis of nanospheres of TiO_2_NPs (size 14–17 nm). The biosynthesized TiO_2_NPs exhibited excellent photocatalytic activity against methyl orange (MO) dye and degraded 99% of MOdye; thus, they can be used in the bioremediation of organic dyes. *Halomonas elongata* (strain IBRC-M 10214), a Gram-negative proteobacteria, was successfully deployed for the green synthesis of TiO_2_NPs and ZnONPs [130]. FTIR and SEM analysis showed the formation of well-dispersed spherical TiO_2_NPs (size 104.63 nm) and ZnO NPs (size 18.11 nm). The antimicrobial activity of both the metal oxide NPs was tested against *S. aureus* ATCC 43300 (Gram-positive) and *E. coli* ATCC 25922 (Gram negative). The ZnONPs exhibited strong antimicrobial activity against both types in comparison to TiO_2_NPs. In a recent report, *Streptomyces* sp. HC1 isolated from soil was used for the synthesis of well-dispersed spherical TiO_2_NPs (size 30–70 nm) that exhibited strong antimicrobial activity against *E. coli* (ATCC 35218) and *S. aureus* (ATCC 29213) and a reasonable antibiofilm activity against *P. aeruginosa* ATCC 27853 [131]. Iron oxide NPs (IONPs) are also important metal oxide NPs which have specific magnetic properties and have applications in imaging, targeted drug delivery, bioremediation and antimicrobial and antifungal activity. The extracellular biofabrication of IONPs (Fe_3_O_4_) has been successfully achieved using *B. subtilis* extracted from soil [132]. SEM analysis confirmed the formation of spherical 60–80-nm-sized NPs of Fe_3_O_4_. In a recent report, the antibacterial, antioxidant and cytocompatibility properties of IONPs biosynthesized using *Proteus vulgaris* ATCC-29905 were successfully analyzed [133]. The spherical IONPs (size 19.23–30.51 nm) showed strong antimicrobial activity against *S. aureus* (methicillin resistant), showed an antioxidant property, inhibited the migration of HT-29 cancer cells and exhibited a strong anticancer property against U87 MG-glioblastoma cancer cells.

#### 2.1.3. Organic Nanoparticles

It has been reported that the extracellular polymeric substances (EPS) of bacteria can be deployed as an effective capping agent and bioreductant for the biofabrication of NPs from metal ions [134,135,136]. For instance, Mehta et al., 2014 used extracellular polymeric substances (EPS) extracted from the marine bacterium *Alteromonas macleodii* for the biofabrication of AgNPs [135]. Various mechanisms involved in the biofabrication of NPs by bacterial cells have been recently reported. It has been proposed that the intracellular or extracellular biosynthesis of NPs is a part of a cellular detoxification mechanism of bacterial cells via an enzymatic reduction which results in confirmatory changes in metal ions solubility [5]. These confirmatory changes in metal ions solubility lead to changing the soluble toxic metal ions to innoxious insoluble NPs. Different biocatalysts involved in the intracellular and extracellular biofabrication of NPs include cellular transporters and oxidoreductase enzymes (such as cysteine desulfhydrase, NADPH-dependent sulfite reductase flavoprotein subunit and NADH-dependent nitrate reductase) [5].

Certain bacterial genera have the specific capability of the biofabrication of some organic nanostructures. For instance, the aerobic acetic bacteria of genus *Gluconacetobacter* efficiently biosynthesized bacterial nanocellulose (3D network of cellulose nanofibrils) which was more crystalline, mechanically stable and purer than nanofibrillated cellulose and nanocrystalline cellulose [24]. Hence, due to its specific properties, bacterial nanocellulose can be used as nanocomposite in biosensoristic applications and as a scaffold for tissue engineering and drug delivery systems and as an antimicrobial agent in biomedical applications [137,138,139]. Another example of an extracellular microbial biopolymer is found in exopolysaccharides, which has applications as a protection agent and as an adhesive of bacterial biofilm. Li et al., 2017 demonstrated that when a nanosized spherical exopolysaccharide was used with the bacteria *Lactobacillus plantarum*-605, it resulted in the rapid biofabrication of monodispersed Ag and AuNPs [23].

### 2.2. Actinobacteria-Mediated Biosynthesis of Nanoparticles

Actinobacteria is a phylum under the bacterial domain which mainly consists of Gram-positive bacteria. These bacteria may be found in aquatic or terrestrial habitats, may decompose dead organic matter or occur as symbionts fixing nitrogen for the plant. Actinobacteria also have the potential to synthesize different biologically active compounds and NPs through extracellular or intracellular routes [4]. However, the extracellular reduction method is mostly preferred for commercial applications. Otari et al., 2012 demonstrated that the enzymes present on the cell wall of *Rhodococcus* NCIM 2891 facilitate in the biomineralization of Ag ions and the intracellular synthesis of AgNPs [140]. The biofabricated AgNPs were 10 nm (average diameter) in size and spherical in shape as revealed from TEM analysis. However, in another finding, Karthik et al., 2014 reported the extracellular biosynthesis of AgNPs using the marine bacterium *Streptomyces* sp. LK-3. They observed that the reduction of Ag ions into stable AgNPs was mediated by NADH-dependent nitrate reductase through an electron transfer reaction [141]. These extracellularly biosynthesized AgNPs showed high antiparasitic activity against *Haemaphysalis bispinosa* and *Rhipicephalus microplus*. In 2016, Abd-Elnaby et al., 2016 isolated 41 actinomycetes from the Suez Gulf of the Red Sea and screened these strains for their capacity to synthesize AgNPs. The study concluded that among all the strains, the marine bacterium *Streptomyces rochei* MHM13 was the most competent in fabricating AgNPs [142]. These AgNPs showed high antibacterial properties against various bacteria, such as *B. subtilis*, *E. coli*, *B. cereus*, *V. uvialis*, *P. aeruginosa* and *S. typhimurium* [142]. Similarly, in a recent study, Buszewski et al., 2018 biofabricated stable AgNPs of spherical shape (8 to 48 nm size) using the acidophilic actinobacteria *Streptacidiphilus durhamensis*. The study concluded that the biofabricated AgNPs showed a higher antimicrobial activity than AgNPs synthesized via conventional methods against *Proteus mirabilis*, *S. aureus* and *P. aeruginosa* [20]. The higher antimicrobial activity of biofabricated AgNPs was due to the stabilization and capping of biofabricated AgNPs with different biologically active molecules. Similarly, Wypij et al., 2018 demonstrated the biosynthesis of spherical polydispersed AgNPs (5–20 nm) using the actinobacterial *Streptomyces xinghaiensis* OF1 strain [143]. The biosynthesized AgNPs along with antibiotics exhibited strong synergetic antibacterial activity against *B. subtilis*, *P. aeruginosa*, *E. coli* and *S. aureus* and antifungal activity against the yeasts *Malassezia furfur* and *Candida albicans*.

In another recent report, Bakhtiari-Sardari et al., 2020 biosynthesized AgNPs using the two actinobacterial strains *Streptomyces* sp. OSIP1 and *Streptomyces* sp. OSNP14 [144]. These cold-tolerant strains produced small AgNPs of 8 nm (OSIP1) and 15 nm (OSNP14) in size, which exhibited strong antibacterial activity against *Pseudomonas aeruginosa* and strong anticancer activity against mouse colorectal carcinoma cells (CT26). These AgNPs, synthesized from different strains, showed similar biological activity although they had different sizes. Gold NPs have also been successfully synthesized using actinobacteria. Ahmad et al., 2003 synthesized uniform-sized AuNPs using the extremophilic actinobacteria *Thermomonospora* sp. and concluded that the enzymatic process of bacterium results in metal ions reduction and the stabilization of newly formed AuNPs [80]. Moreover, it was observed that *Thermomonospora* sp. can also fabricate the monodisperse AuNPs in extreme biological conditions, such as elevated temperature and high alkalinity [145]. In another study, Ahmad et al., 2003 reported that *Rhodococcus* sp., an alkalotolerant bacteria, can tolerate high metal ion concentrations (10^−3^ M HAuCl_4_) during the biofabrication of AuNPs. The authors reported that *Rhodococcus* sp. Produced monodisperse AuNPs (5–15 nM) intracellularly. The cells of bacterium continued growth even in the higher Au ion concentration and after the biofabrication of AuNPs [146]. He et al., 2007 reported that at lower concentrations of metal ions (2.5 × 10^−4^ M HauCl_4_), *Rhodopseudomonas capsulata* biosynthesized spherical AuNPs (size 10–20 nm), while at higher concentrations, it produced nanowires (diameter between 50 and 60 nm) [147].

Actinobacteria have also been successfully deployed for the synthesis of other NPs [148,149]. For example, Ranjitha and Rai (2017) documented the first report on the extracellular biosynthesis of AuNPs using actinobacteria *Streptomyces griseoruber* isolated from the soil. Gold NPs of 5–50 nm in size produced via bioreduction exhibited strong catalytic activity for the decomposition of methylene blue [148]. In an interesting study, Hassan et al., 2018 biosynthesized CuNPs using the endophytic actinobacterial strain *Streptomyces capillispiralis* Ca-1, isolated from the medicinal plant *Convolvulus arvensis* L. The biofabrication of CuNPs was affirmed by a visual change in the color of biomass filtrate from light blue to greenish brown [149]. TEM analysis confirmed the production of spherical monodispersed CuNPs having a size of 3.6–59 nm. FTIR analysis showed that the various bioactive functional groups present in actinobacteria help in the stabilization and formation of CuNPs. The biofabricated CuNPs exhibited good antimicrobial activity and antifungal activity.

## 3. Fungi- and Yeast-Mediated Biosynthesis of Nanoparticles

### 3.1. Metal Nanoparticles

Biofabrication of NPs using fungi is another biological method of NP synthesis. Fungi have a high cell wall binding potential with metal ions and have a higher potential to tolerate metal concentrations; hence, fungi can yield a higher number of NPs than bacterial cells [18]. The production of NPs using fungi is more efficient and inexpensive than bacteria, as fungi have a higher tendency to accumulate metals. Moreover, the treatment of biomass and downstream processing of NPs is simpler in the fungi-based biosynthesis of NPs. Therefore, fungi have been widely studied for the synthesis of different NPs, such as silver, gold, etc. In the past few years, it has been observed that the maximum work has been carried out on the extracellular fabrication of NPs using fungi, as it avoids the use of detergents; physical factors, such as ultrasound; and the doping of intracellular components, such as proteins, fats, nucleic acids, etc. [4]. For instance, Mishra et al., 2014 demonstrated the extracellular biosynthesis of AuNPs using the fungi *Trichoderma viride* and *Hypocrea* *lixii* and also described the effect of pH and temperature on the biosynthesis of AuNPs [150]. They observed that the cell-free extract of *T. viride* fabricated the AuNPs within 10 min of reaction at 30 °C, while *H. lixii* synthesized the AuNPs at 100 °C. The size of the newly synthesized AuNPs ranged between 20 and 30 nm. It was also observed that *T. viride* synthesized AuNPs at a higher pH (pH 7 and 9) but failed to synthesize NPs at pH 5 at room temperature. Although *H. lixii* failed to fabricate NPs at room temperature in the same pH range, it synthesized NPs when the reaction mixture was boiled at 100 °C. The biosynthesized gold NPs exhibited strong antimicrobial and biocatalytic activities. Similarly, Metuku et al., 2014 fabricated AgNPs using the fungi *Schizophyllum radiatum*. It was observed that the fungi produced AgNPs of 10 to 40 nm in size via extracellular biomineralization, which showed strong antimicrobial properties against both Gram-positive and -negative bacteria [151].

Factors such as medium pH, reaction time and ionic concentrations largely influence the yield and size of NPs. Hence, many studies have been conducted to study the effect of these parameters on the biofabrication of NPs. For example, Bhargava et al., 2016 investigated the influence of ionic concentration and pH on the biosynthesis of AuNPs using the fungi *Cladosporium oxysporum* and reported the highest yield of AuNPs with pH 7.0, 1 mM ionic concentration and 1:5 biomass to water ratio [152]. Similarly, Rajput et al., 2016 also studied the effect of pH, temperature and isolate selection on the biosynthesis of AgNPs using 12 different isolates of the fungi *Fusarium oxysporum.* It was found that different medium pH (3, 5, 7 and 9) affects the shape of AgNPs using *Fusarium oxysporum* 405. At pH 3.0, AgNPs of triangular, spherical, rod and other irregular shapes were produced, while at pH 5 and 7, monodisperse and mostly spherical NPs were formed [153]. A further increase in pH up to 9 led to the fabrication of a combination of oblong and spherical NPs. The change in pH of the medium led to the formation of different sizes of NPs because the change in pH influences the basic or acidic nature of amino acids, which are involved in the fabrication of NPs [153,154,155]. Birla et al., 2013 studied the effect of temperature on the biosynthesis of AgNPs using *Fusarium oxysporum* and reported that a temperature of 40 to 60 °C is optimal for the synthesis of AgNPs [155]. Similarly, Rajput et al., 2016 also studied the effect of different temperatures on the quantity of AgNPs produced from *F. oxysporum* 405 and found that the maximum NPs were formed at a temperature between 50 to 70 °C, while minimum AgNP formation was observed at 25 °C. The incubation temperatures also affected the size of newly synthesized AgNPs. At the optimum temperatures (50 to 70 °C), small sized NPs (10 nm) were formed, while at 25 °C, AgNPs were more than 50 nm in diameter [153]. The biofabrication of other important NPs apart from AgNPs has also been explored using various fungal strains; for example, Kitching et al., 2016 demonstrated the biosynthesis of spherical and 16–19-nm-sized AuNPs from cell surface proteins isolated from *Rhizopus oryzae*. These biofabricated AuNPs were found to be stable and hemocompatible and therefore can be used for biocatalytic and biomedical applications [156].

In another study, El Domany et al., 2018 demonstrated the extracellular biofabrication of 10–30-nm-sized AuNPs using the edible fungi *Pleurotus ostreatus* and reported that the fabrication of AuNPs significantly affected by the salt concentration, temperature, pH and incubation time [157]. In this study it was reported that the rate of biofabrication of AuNPs was directly proportional to the incubation time, salt concentration and temperature. As the incubation time, salt concentration and temperature were increased to 12–48 h, 1–5 mM and 30–40 °C, respectively, the rate of AuNP biofabrication also increased. However, the maximum AuNPs were synthesized at pH 3.0. Neethu et al., 2018 demonstrated the green synthesis of AgNPs from the fungi *Penicillium* *polonicum*, isolated from the marine green alga *Chetomorpha antennina*, and concluded that the biosynthesized AgNPs showed strong antibacterial activity against the biofilm-forming multidrug-resistant bacterium *Acinetobacter baumanii* [158]. In a recent report, Clarance et al., 2019 reported the biosynthesis of 40–45-nm-sized AuNPs from the endophytic fungi *Fusarium solani* ATLOY-8, isolated from the plant *Chonemorpha fragrans*. The biosynthesized AuNPs were highly stable and showed good anticancerous activity against HeLa and MCF-7 cell lines [159]. Similarly, Munawer et al., 2020 reported the extracellular green synthesis of 5–10-nm-sized spherical AuNPs from the fungi *Cladosporium* sp., extracted from the medicinal plant *Commiphora wightii*, which showed excellent antiproliferative activity against the breast cancer cell line MCF-7 [160]. Similarly, Ramos et al., 2020 reported the extracellular biosynthesis of AgNPs from the fungi *Trichoderma* spp., extracted from seeds of plant *Bertholletia excelsa* (Brazil nut), and concluded that the biofabricated AgNPs had superior antibacterial activity against Gram-negative bacteria [161].

Yeasts have the potential to survive in a high concentration of metal ions and have the capability to deposit a high amount of metal ions from a medium [14]. This feature of yeast has been used by different researchers for the green synthesis of NPs [35,162,163]. For instance, Apte et al., 2013 exploited the marine yeast *Yarrowia lipolytica* for the biosynthesis of AgNPs and concluded that melanin (a brown pigment found in yeast) was associated with green synthesis of AgNPs [162]. In a different study, Waghmare et al., 2015 demonstrated the extracellular biofabrication of 20–80-nm-sized AgNPs from the yeast strain *Candida utilis* NCIM 3469 and concluded that these biofabricated AgNPs exhibited a good antibacterial property against disease-causing strains of the bacteria *P. aeruginosa*, *E. coli* and *S. aureus* [35]. Similarly, AgNPs of 2–10 nm in size with strong antibacterial activity against *K. pneumonia* and *S. aureus* were biosynthesized from a yeast strain of *Candida lusitaniae* isolated from the gut of a termite [164]. Zhang et al., 2016 demonstrated, for the first time, the green synthesis of AuNPs from the yeast *Magnusiomyces ingens LH-F1*. They reported the biosynthesis of AuNPs of different sizes (hexagon, spheres and triangles) that showed strong reducing properties against nitrophenols [163]. Genetically engineered yeast has also been used for the fabrication of metal NPs. In the recent past, Elahian et al., 2017 successfully used the genetically altered yeast strain *Pichia pastoris* for the fabrication of AgNPs [165]. In this study, it was demonstrated that the genetically altered yeast strain *Pichia pastoris* transformed with an upregulated metal resistant gene from *Mucor racemosus* produced cytochrome b5 reductase enzyme, which reduces the metal ions and produces uniform (size 70–180 nm) and stable AgNPs. In another study, Sriramulu and Sumathi (2018) reported the biosynthesis of PdNPs from *S. cerevisiae*. The newly biosynthesized hexagonal PdNPs of 32 nm in size exhibited good catalytic activity against textile azo dye and degraded 98% dye under UV light within 60 min of exposure [166].

### 3.2. Metal Oxide Nanoparticles

Like bacteria, fungi and yeast have also been explored for the synthesis of various important metal oxide NPs. Aluminum oxide NPs (Al_2_O_3_NPs) are important metal oxide NPs which have significant antimicrobial properties against multidrug-resistant bacteria [167]. In a unique report, Al_2_O_3_NPs were biosynthesized using *Colletotrichum* sp. and were made functional using plant oils extracted from *Citrus medica* and *Eucalyptus globules*. The nanoactivated oils exhibited effective antimicrobial activity against food spoiling pathogens [168]. In an interesting study, ZnONPs were extracellularly biofabricated using *Aspergillus terreus* AF1 and were tested for their biomedical properties [169]. The *A. terreus* AF1 effectively reduced the ZnO to ZnONPs, and FTIR analysis confirmed that the proteins secreted by *A. terreus* AF1 acted as a capping agent of the ZnONPs. These green-synthesized spherical ZnONPs (size 10–45 nm) exhibited strong antimicrobial activity against *P. aeruginosa*, *S. aureus*, *B. subtilis* and *E. coli* and a reasonable cytotoxic effect against Vero and Caco cell lines. Cotton fabric was blended with the ZnONPs, and the deposition of ZnONPS on the cotton fabric was confirmed by SEM. It was observed that this blend exhibited moderate antibacterial activity against *E. coli* and *P. aeruginosa* and also blocked the passage of UVA (76.3%) and UVB (85.4%) in comparison to untreated cotton fabric. In another important work, Mohamed et al., 2019 studied the effect of fungal strains on the morphology and bioactivity of ZnONPs. In this study, ZnONPs were synthesized using two fungi, *A. niger* strain (G3-1) and *F. keratoplasticum* strain (A1-3), isolated from soil. It was reported that ZnONPs of different morphology were synthesized using different fungal strains [170]. Hexagonal ZnONPs (size 10–42 nm) were fabricated using the *F. keratoplasticum* strain (A1-3) while nanorods (size 8–32 nm) were synthesized using the *A. niger* strain (G3-1). It was also reported that nanorod ZnONPs were more effective against both Gram-positive bacteria (*B. subtilis* and *S. aureus*) and Gram-negative bacteria (*E. coli* ATCC 8739 and *P. aeruginosa* ATCC 9027) than hexagonal ZnONPs. Hence, the bioactivity of NPs was governed by their shape and size due to the variation in physical and chemical properties. Recently, in a unique report, Sumanth et al., 2020 fabricated ZnONPs using *Xylaria acuta* isolated from the plant *Millingtonia hortensis* L.f. The hexagonal ZnONPs (size 34–55 nm) showed strong antimicrobial properties against the bacteria *P. aeruginosa, E. coli*, *S. aureus* and *B. cereus* and the fungi *Cladosporium cladosporioides* and a good anticancer property against human MDA-MB 134 mammary gland carcinoma cells [171]. ZnO and CuONPs also act as good antibiofilm, antibacterial and antifungal agents against multidrug-resistant microbes and hence can be used as potent antimicrobial nanomaterials [172]. In a recent report, the efficiency of ZnONPs (size 9–35 nm) and CuONPs (size 10.5–59.7 nm) fabricated using *P. chrysogenum* as antibiofilm and antibacterial agents was tested [173]. It was reported that CuONPs showed higher antibacterial activity than ZnONPs against both Gram-positive and Gram-negative bacteria. In an interesting recent study, mycogenic CuONPs have been tested for their insecticidal and growth regulator activity in wheat (*Triticum aestivum* L.) storage [174]. Spherical CuONPs (size 14–47.4 nm) were synthesized using *A. niger* G3-1 and characterized using SEM, TEM, XRD and FTIR. The insecticidal property of CuONPs was tested during the storage of wheat, and it was reported that NP treatment leads to the mortality of grain insects *Sitophilus granaries* (55–94.4%) and *Rhyzopertha dominica* (70–90%). The CuONPs 50 ppm dose also significantly stimulated the growth and physiology of wheat grain germination. In a unique report, CuO/ZnO nanocomposites were biofabricated using cell filtrate of *Penicillium corylophilum* As-1, and their photocatalytic activity was assessed against methylene blue dye [175]. It was reported that nanocomposites having CuO and ZnO in a ratio of 20:80 (size 10–55 nm) degraded 97% of methylene blue dye. TiO_2_ is another important metal oxide which is naturally obtained from rutile, brookite or anatase minerals and has an array of applications in pharmaceuticals, photocatalytic activity, food, bioremediation, agriculture and cosmetics [125,176]. The photocatalytic and antimicrobial activity of spherical TiO_2_ NPs (anatase, size 6.7 nm) fabricated using Baker’s yeast was tested against methylene blue dye and the Gram-positive bacteria *C. albicans* [176]. TiO_2_ NPs efficiently degraded the methylene blue and significantly controlled the growth of *Candida albicans*. In a unique study, TiO_2_NPs synthesized using *T. viride* were used as biopesticides. The pupicidal, larvicidal and antifeedant properties of spherical TiO_2_NPs (size 60–86.67 nm) were tested against *Helicoverpa armigera*. It was reported that in a filter paper assay, *Trichoderma viride*-mediated TiO_2_NPs (100 ppm) showed significant biopesticidal activity and enhanced the mortality rate of *H. armigera* up to 100%, while they did not exhibit any toxic effect against *Eudrilus eugeniae* (earthworm) [177]. Recently, the anticancer and antibacterial activity of TiO_2_NPs synthesized using *Pleurotus djamor* (an edible mushroom) has been studied, and it has been reported that TiO_2_NPs (31 nm) showed strong anticancer activity against A549 cancer cells and significant antibacterial activity against *C. diphtheria*, *S. aureus* and *P. fluorescens* [178]. IONPs have immense capabilities in biomedicine, environment, life science, agriculture, biosensors and storage [179]. Bhargava et al., 2013 biosynthesized cubic IONPs (size 60–70 nm) deploying *Aspergillus japonicus* (AJP01). FTIR data affirmed that the biosynthesized IONPs were stabilized by the fungal proteins [180]. Recently, Mahanty et al., 2019 synthesized IONPs (size 2–16 nm) using manglicolous fungi (*Fusarium incarnatum*, *Trichoderma asperellum* and *Phialemoniopsis ocularis*) isolated from the waterlogged Sundarban (India). The role of these IONPs was also studied in the bioremediation of water containing chromium, and it was reported that the IONPs exhibited an excellent chemisorption property towards Cr(VI) with a 4.62 mg/g adsorption capacity [181,182]. In a novel attempt, Vijayanandan et al., 2018 synthesized cobalt oxide NPs (CoONPs) using *Aspergillus nidulans*. The biosynthesized spinel CoONPs (size 20.29) were used in energy storage and showed a promising capacitance of 389 F/g [183]. From the above reports, it can be concluded that fungi and yeast have immense potential for the green synthesis of NPs.

## 4. Algae-Mediated Biosynthesis of Nanoparticles

### 4.1. Metal Nanoparticles

Algae are autotrophic organisms and can easily grow on minimal medium supplements. Algae cells have different secondary metabolites and many biologically active compounds which act as capping agents during NP synthesis and make the algal cell a unique “nanofactory” for the synthesis of various NPs [184]. Dhas et al., 2014 extracellularly biofabricated AgCl NPs using the marine alga *Sargassum* *plagiophyllum*. TEM analysis confirmed the synthesis of spherical AgCl NPs of 21–48 nm in size [185]. The biofabricated NPs of AgCl showed reasonable antibacterial activity against *E. coli*. Similarly, Edison et al., 2016 demonstrated the biosynthesis of highly stable AgNPs using an extract of the marine alga *Caulerpa racemosa* (green algae). The newly synthesized AgNPs were 25 nm in size and exhibited excellent catalytic activity against methylene blue [186]. In another study, da Silva Ferreira et al., 2017 demonstrated the extracellular biosynthesis of AgCl NPs using a dried biomass of the green unicellular alga *Chlorella vulgaris*. FTIR investigation of biosynthesized AgCl NPs revealed that the proteins of alga were the chief capping agent engaged in the stabilization and formation of AgCl NPs [187]. TEM investigation showed that most of the AgCl NPs were spherical in shape and 9.8 nm in size. The newly synthesized AgCl NPs exhibited strong antimicrobial activity against the pathogenic bacteria *S. aureus* (Gram-positive) and *K. pneumoniae* (Gram-negative). Kim et al., 2018 also reported the extracellular biosynthesis of AgNPs using a cellular extract of the alga *Laminaria japonica* and concluded that the rate of the biosynthesis of AgNPs was significantly enhanced when *L. japonica* cellular extract was incubated with AgNO_3_ at 120 °C [188]. In a recent report, Fatima et al., 2020 demonstrated the biosynthesis of spherical AgNPs using the red alga *Portieria hornemannii* [189]. The AgNPs exhibited good antimicrobial activity against the bacteria *Vibrio parahaemolyticus*, *Vibrio anguillarum*, *Vibrio alginolyticus* and *Vibrio harveyii*. In another recent report, Bhuyar et al., 2020 analyzed the biosynthesis of 25–60-nm-sized AgNPs using the marine microalga *Padina* sp. [190]. The AgNPs were uniform in size and exhibited strong antimicrobial activity against the pathogenic bacteria *P. aeruginosa* and *S. aureus*.

Reports are also available on the biosynthesis of AuNPs using algae. Senapati et al., 2012 reported the intracellular biosynthesis of AuNPs of 5–35 nm in size using the alga *Tetraselmisko chinensis* [191], while Singh et al., 2013 demonstrated the extracellular biosynthesis of 53–67-nm-sized AuNPs using the macroalga *Padina gymnospora* [192]. Dahoumane et al., 2016 demonstrated the biosynthesis of AuNPs using the microalga *Euglena gracilis*. The AuNPs were synthesized using cells of *E. gracilis*. It was observed that this microalga exhibited seven to eight times faster growth, which improved the kinetics, the colloidal stability and the yield of the biofabricated AuNPs (with variable shapes, including round, hexagons, pentagons, triangles and truncated triangles) [193]. In a different study, Ramakrishna et al., 2016 demonstrated the extracellular biosynthesis of AuNPs by employing a cellular extract of the brown algae *Turbinaria conoides* and *Sargassum tenerrimum*. High-resolution TEM investigation showed the biosynthesis of nearly spherical AuNPs of 27–35 nm in size [194]. The biofabricated NPs exhibited a good degradation ability against organic dyes and aromatic nitro compounds. Similarly, González-Ballesteros et al., 2017 also reported the extracellular synthesis of AuNPs using an extract of the brown macroalga *Cystoseira baccata*. The spherical AuNPs were stable and 8.4 nm in size. The AuNPs exhibited an acceptable cytotoxic effect towards the normal primary neonatal dermal fibroblast cell line PCS-201-010 and colon cancer cell lines Caco-2 and HT-29 [195].

PdNPs have also been synthesized using algal cellular machinery. Momeni and Nabipour (2015) demonstrated the biosynthesis of PdNPs using the marine alga *Sargassum bovinum* [196]. In an interesting study, Arsiya et al., 2017 reported the extracellular quick biosynthesis of PdNPs using a cellular extract of the green alga *Chlorella vulgaris*. The PdNPs were synthesized within 10 min of incubation with the extract of *C. vulgaris*. TEM analysis showed the biofabrication of uniform, circular, 5–20-nm-sized PdNPs [197]. In another study, Sayadi et al., 2018 demonstrated the extracellular biosynthesis of PdNPs using a cellular extract of the alga *Spirulina platensis*. TEM results revealed that the PdNPs were spherical in shape and were 10–20 nm in size. The biosynthesized PdNPs were successfully used in the bioremediation of lead [198].

### 4.2. Metal Oxide Nanoparticles

As discussed above, the microbial cellular factory has been successfully used for the synthesis of metal and metal oxide NPs. Algae have also been deployed for synthesizing different metal oxide NPs. Rajeshkumar (2018) extracellularly biofabricated ZnONPs using two marine brown algae, *Turbinaria conoides* and *Padina tetrastromatica*. The crystalline nature of newly synthesized ZnONPs was characterized by using XRD and SEM [199]. The biosynthesized ZnONPs exhibited strong antimicrobial activity against fish pathogens. In another similar study, Sanaeimehr et al., 2018 reported the extracellular biosynthesis of ZnONPs using an extract of the alga *Sargassum muticum*. The biofabricated ZnONPs exhibited strong antiapoptotic and antiangiogenic activity toward human liver cancer cell lines [21]. Khalafi et al., 2019 demonstrated the extracellular biosynthesis of ZnONPs using an extract of the green microalgae *Chlorella*. The biosynthesized ZnONPs were characterized using SEM and TEM. These monodispersed hexagonal-shaped ZnONPs had an average size of 20 nm and exhibited reasonable remediation properties against organosulfur pollutants [200]. In a unique study, ZnO nanorods were synthesized using carbohydrates extracted from the green algae *Chlorella vulgaris*. The ZnO nanorods (length 150 nm and width 21 nm) showed an excellent screening effect against UVA and UVB and a strong antibacterial activity against both Gram-positive and Gram-negative bacteria [201]. In a recent report, ZnONPs have been biosynthesized using the blue green algae *Arthrospira platensis* [202]. The ZnONPs were characterized using FTIR, XRD and TEM. The spherical ZnONPs (size 30–55 nm) exhibited significant antimicrobial activity against *E. coli*, *B. subtilis*, *P. aeruginosa*, *S. aureus* and *C. albicans*. In a specific study, Sharma et al., 2018 biosynthesized TiO_2_NPs and a TiO_2_-graphene oxide (GO) nanocomposite using *Chlorella pyrenoidosa* (a green alga). The XRD and SEM analysis confirmed the formation of spherical TiO_2_NPs (size 50 nm) and a sheet-like TiO_2_-GO nanocomposite. The photocatalytic activity of both nanostructures was tested and compared against crystal violet (CV) dye, and it was reported that the TiO_2_-GO nanocomposite more efficiently degraded the CV than the TiO_2_NPs [203]. El-Kassas et al., 2016 reported the green synthesis of IONPs (Fe_3_O_4_) using two brown algae (seaweeds), *Sargassum acinarium* and *Padina pavonica* [204]. TEM analysis confirmed the formation of IONPs of 21.6–27.4 nm using *S. acinarium* and 10–19.5 nm using *P. pavonica*. The green-synthesized IONPs were then encapsulated in calcium alginates beads (CAB) and were used for the bioremediation of lead (Pb). It was reported that the IONPs-CAB synthesized using *S. acinarium* absorbed 78% of Pb while IONPs-CAB from *P. pavonica* removed 91% Pb. The study affirmed the role of NPs in bioremediation. The algal cells have several secondary metabolites and many biologically active compounds which act as capping agents during NP synthesis and make them unique “nanofactories” for the synthesis of various NPs [184].

## 5. Virus-Mediated Biosynthesis of Nanoparticles

Viruses are covered with capsid proteins of nanoscale structures (20–500 nm), which provide appropriate tenets to metallic ions to interact with virus machinery [205]. The capsid protein can be modified via genetic engineering for the synthesis of nanomaterials, such as nanocomposites and nanoconjugates of metal NPs, for the treatment of cancer and targeted drug delivery [4]. Many reports are available where viruses have been successfully deployed for the biosynthesis of NPs. For instance, M13 bacteriophage has been successfully used as a template for the fabrication of semiconductor NPs of cadmium sulphide (CdS) and zinc sulphide (ZnS) [206]. Plant viruses are stable, easy to cultivate and nonpathogenic to animals and humans. Hence, plant viruses can also be deployed for the fabrication of NPs for use in humans and animals. For example, Slocik et al., 2005 synthesized AuNPs using cowpea chlorotic mottle viruses as a template [207]. Similarly, Kobayashi et al., 2012 demonstrated the green synthesis of uniform shaped AuNPs (size 5 nm) using the tobacco mosaic virus (TMV) [205]. Cao et al., 2014 synthesized nanocarriers using the red clover necrotic mosaic virus (RCNMV), which were successfully tested for the regulated delivery of the drug doxorubicin for the treatment of cancer [208]. Similarly, Czapar et al., 2016 synthesized hollow nanotubes with a polyanionic interior surface using the tobacco mosaic virus (TMV) for the targeted delivery of the anticancer drug phenanthriplatin. In this study, the one-step mechanism for the loading of phenanthriplatin and its delivery using TMV nanotubes was successfully demonstrated in a mouse model [209]. In a similar report, Le et al., 2017 used nanocarriers synthesized through the potato virus X. They synthesized elongated lamentousnanocarriers for the targeted delivery of the drug doxorubicin for cancer treatment. These virus-mediated nanocarriers showed an increased penetration power in the tumor compared with spherical NPs [210]. In another study, Chen et al., 2018 reported the biosynthesis of the hepatitis E virus nanoparticle (HEVNP) using the hepatitis E virus capsule. In this study the HEVNP was coupled with a labeled near-infrared (NIR) fluorescence dye and breast cancer cell-specific ligand, LXY30. It was concluded that these biosynthesized HEVNPs could be deployed as multifunctional delivery carriers for tumor imaging, tissue targeting and therapeutic delivery [211].

In a recent study, Thangavelu et al., 2020 synthesized Au-Ag composite semiconductor NPs using the plant pathogenic squash leaf curl China virus (SLCCNV) [212]. The virus was used as a nanobiotemplate (32 nm) to synthesize the Au-Ag nanomaterials. The Au-Ag nanomaterial was tested for electrical conductivity using Keithley’s picoammeter and the “lab on a chip” system. It was concluded that the hybrid nanomaterial (Au-Ag) showed excellent semiconductive properties and good biocompatibility for biomedical applications. In the aforementioned reports, viruses have been successfully used as a template for the synthesis of NPs; however, virus-mediated NP synthesis still has certain limitations, such as large-scale application, biocompatibility, the requirement of a host for protein expression and under-defined methods for the synthesis of NPs.

## 6. Applications of Nanoparticles

**Figure 3:**
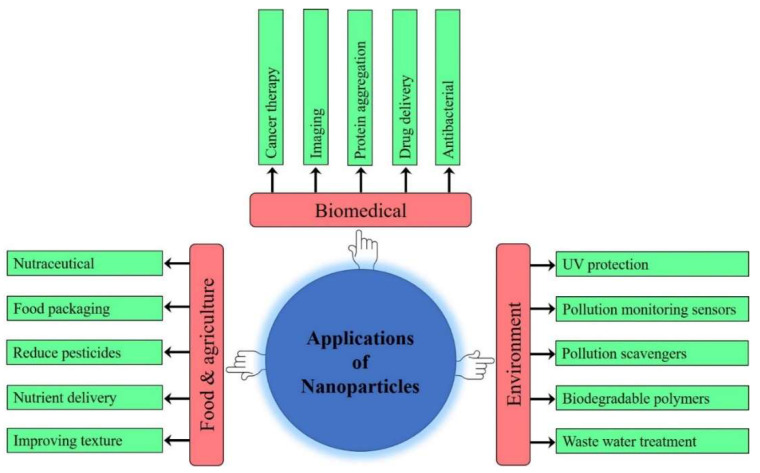
Applications of NPs in different fields.

Due to their unique size, shape, structure and specific biological, physical and chemical properties, nanoparticles are suitable candidates for various applications in different fields. (1) Biomedical: drug delivery, disease diagnosis, cancer therapy, antibacterial activity, etc. (2) Food and agriculture: nutraceutical, food packaging, pesticides, nutrient availability, nanobiosensors for crop protection, nanoformulations of agrochemicals, nanodevices for the genetic engineering of plants, animal health, postharvest management, etc. (3) Environment: bioremediation, water treatment, biodegradable polymers, pollution monitoring sensors, UV protection, etc. [28,213] (Figure 3). The applications of microbially synthesized NPs in different fields are discussed in the following section of this review.

### 6.1. Biomedical Applications of Nanoparticles

Pathogenic microbes develop resistance against antibiotics via changing their metabolic pathways and target sites [214]. Moreover, these microbes develop more pathogenicity and become resistant to antibiotics due to their continuous exposure [15]. Hence, it is essential to develop certain alternative, unconventional, powerful antimicrobial agents which are cheaper and safer. Biofabricated NPs have a larger surface area and smaller size. These properties allow NPs to efficiently interact with the microbial cell membrane and penetrate the cells to interfere with the metabolic pathways and DNA replication [15]. The antibacterial activities of different NPs have been reported in many studies.

Sondi and Salopek-Sondi (2004) studied the possible process of the antibacterial action of NPs, which involves the destruction of the cell membrane and cellular machinery via the formation of pits which culminate in the death of bacterial cells [215]. In addition to this, if the NPs are used along with traditional antibiotics, the antibacterial efficiency of NPs can be enhanced. For example, Banuet al., 2011 studied the combined effect of AgNPs (biofabricated using bacterium *R. stolonifer*) and antibiotics (ciprofloxacin, nitrofurantoin and carbenicillin) against bacterial species of the family Enterobacteriaceae (ESBL-strains). The decreasing order of the antibacterial activity was: nitrofurantoin (50% efficacy) > carbenicillin (33.56% efficacy) > ciprofloxacin (30.53% efficacy) [216]. Sunkar and Nachiyar (2012) studied the antibacterial activity of AgNPs synthesized from *Bacillus cereus* (using the agar-well diffusion method) against the disease-causing bacteria *Staphylococcus aureus*, *Klebsiella pneumonia*, *Pseudomonas aeruginosa*, *Escherichia coli* and *Salmonella typhi*. It was observed that AgNPs produced larger zones of inhibition in comparison to standard antibiotics (amoxicillin, streptomycin and ofloxacin) [68]. Singh et al., 2015 studied the synergetic effect of AgNPs obtained from *Brevibacterium frigoritolerans* in combination with the antibiotics oleandomycin, novobiocin, vancomycin, rifampicin, lincomycin and penicillin G [217]. They reported that the antimicrobial efficacy of all the antibiotics was enhanced against the disease-causing strains of *Candida albicans*, *Bacillus anthracis*, *Vibrio parahaemolyticus*, *Escherichia coli*, *Salmonella enterica* and *Bacillus cereus* when used with AgNPs. Elbeshehy et al., 2015 fabricated AgNPs using three strains of *Bacillus licheniformis*, *B. persicus* and *Bacillus pumilus* and tested the antimicrobial activity of these AgNPs against the human pathogens *Candida albicans* ATCC 1021, *Escherichia coli* ATCC 25922, *Staphylococcus epidermidis* ATCC 1228, *Aspergillus flavus*, *Shigella sonnei* ATCC25931, *Streptococcus bovis* ATCC 49147, *Pseudomonas aeruginosa*, *Klebsiella pneumonia* ATCC 700603 and *Staphylococcus aureus* (methicillin-resistant strain) ATCC 43330 [218]. The maximum antimicrobial activity was observed with the AgNPs synthesized using *B. licheniformis*.

Mahmoud et al., 2016 biosynthesized AgNPs from *Bacillus pumilus* and studied the antimicrobial activity of these NPs against the pathogenic bacteria *S. aureu, S. bovis*, *S. sonnei*, *E. coli*, *S. typhimurium* and *K. pneumonia* [219]. Similarly, Roychoudhury et al., 2016 synthesized AgNPs using the bacterium *Lyngbya majuscule* (CUH/Al/MW-150) and found that these AgNPs showed effective antibacterial activity against *P. aeruginosa*. These AgNPs were also observed to act as effective antiproliferative agents against leukemic cells and the REH cell line [42]. Al-Dhabi et al., 2018 observed that AgNPs synthesized from *Streptomyces* sp. *Al-dhabi-87* showed strong antibacterial activity against *S. epidermidis*, *E. faecalis*, *B. subtilis* and the multidrug-resistant *S. aureus* strain [31]. There are many reports available regarding the application of AgNPs as antimicrobial agents. However, only few reports are available regarding the use of other NPs as antibacterial agents. For instance, the strong antibacterial activity of AuNPs extracellularly synthesized from *Nocardiopsis* sp. MBRC-48 has been reported against *C. albicans* and *S. aureus* [50]. Malarkodi et al., 2013 studied the antimicrobial efficacy of titanium oxide NPs (TiO2 NPs) obtained from *Planomicrobium* sp. against the Gram-negative bacterium *K. planticola*, Gram-positive bacterium *B. subtilis* and fungi *A. Niger*. In this study, it was observed that the TiO2NPs exhibited the maximum inhibition against both the bacteria at 0.4 ppm concentration in comparison to the antibiotic Kanamycin, while the fungal growth was maximally inhibited at a 1mL concentration of TiO2NPs [220].

There are also reports on the antibacterial activities of zinc oxide NPs against many disease-causing bacteria (Pseudomonas alcaligenes, Proteus mirabilis, Enterobacter aerogenes, Streptococcus pyogenes, Pseudomonas aeruginosa, Salmonella typhimurium, Staphylococcus epidermidis, Proteus vulgaris, Shigella flexneri, Klebsiella pneumonia, Bacillus subtilis and Enterococcus faecalis) [221,222,223]. In a recent report, Jain et al., 2020 biosynthesized ZnONPs using the zinc-resistant bacterium Serratia nematodiphila and, in vitro, tested the antimicrobial activity of ZnONPs against Xanthomonas oryzaepv. oryzae [224]. Xanthomonas oryzaepv. oryzae is resistant to penicillin, while the ZNONPs clearly inhibited the growth of this bacterium.

### 6.2. Role of Nanoparticles in Drug Delivery and Diagnostics

Currently, cancer, which is characterized by the uncontrolled growth of cells, is one of the most threatening diseases. Traditional methods (chemotherapy, radiation and surgery) for curing cancers are not free from ill effects. Moreover, targeted drug delivery to the affected organ and early diagnostic of this devastating disease is still in its infancy [225]. Therefore, it is crucial to develop certain alternative methods of diagnosis and treatment of this disease. It has been reported that nanomedicines can be successfully deployed for the diagnosis of a tumor and its treatment via targeted drug delivery [226]. For example, Borseet al., 2015 studied the in vitro anticancer efficacy of biofabricated PtNPs obtained from *Saccharomyces boulardii* against MCF-7 and A431 cell lines [227]. Nanomedicines have been used against different types of cancer cell lines. Breast cancer has a high reoccurrence rate in women. Ortega et al., 2015 tested the efficacy of AgNPs biosynthesized from *Cryptococcus laurentii* and observed that biofabricated AgNPs showed effective anticancerous and antitumor properties against cancer and breast cancer cell lines [140]. Biosynthesized SeNPs also showed anticancer activity. Ahmad et al., 2015 tested biofabricated Se nanorods (SeNrs) from the *Streptomyces bikiniensis* strain EssamA-1 against human cancer lines and found that these nanorods lead to the mortality of Hep-G2 and MCF-7 cancer cells [47]. Ramya et al., 2015 observed that SeNPs extracellularly fabricated from the bacterium *Streptomyces minutiscleroticus* M10A62 showed considerable antiproliferative activity against HepG2 and HeLa cell lines [41]. El-Batal et al., 2015, studied the efficacy of AuNPs against human liver (HEPG-2) and breast carcinoma (MCF7) cell lines and observed that AuNPs induce apoptosis of mitochondria, cytokinesis detention, impairment of DNA and degradation of nuclei in cancer cell lines [228]. Thus, there are many reports where NPs have been deployed for their anticancerous role in controlled conditions. However, factors such as toxicity, doses and host immune response during treatment are still required to be addressed prior to their commercialization.

Nanomaterials can be effectively used as a vehicle for targeted drug delivery and are a very useful tool in enhancing the bioavailability, stability and bioactivity of drugs. Major drug delivery nanomaterials include liposomes, nanospheres, polymeric micelles, water-soluble polymers, nano emulsions and NP-coated natural antibodies [229,230]. Nanomaterials as drug delivery vehicles have many advantages over traditional methods. Targeted drug delivery can reduce the risk of side-effects caused by the toxicity of drugs in patients. Kundu et al., 2014 studied the efficacy of ZnONPs biofabricated from *Rhodococcus pyridinivorans* as nanovehicles for anthraquinone [231]. The concentration-dependent cytotoxicity of ZnONPs loaded with anthraquinone was observed against HT-29 colon carcinoma cells, and it was concluded that ZnONPs loaded with anthraquinone could be deployed for targeted drug delivery.

Nanomaterials with hydrophilic properties can increase the absorption of drugs and enhance the cytotoxicity of drugs via better diffusion. Kumar et al., 2008 tested biofabricated AuNPs coupled with the drug doxorubicin against HEK293 cancer cell lines and found that the drug coupled with AuNPs more quickly diffused into HEK293 cancer cell lines [232]. Syed et al., 2013 also observed similar results using AuNPs coupled with the drug doxorubicin against hepatic cancer cells [145]. Khan et al., 2014 demonstrated that the anticancer drug taxol coupled with biosynthesized gadolinium oxide NPs as a drug delivery system could be used effectively in the treatment of cancers [233].

The conventional methods of disease diagnosis are laborious, costly and time consuming. NPs can offer cost effective, fast, specific and accurate detection of pathogens as well as chronic diseases. Nanomaterials, such as magnetic NPs, fluorescent NPs (such as quantum dots and dye-loaded NPs) and metallic NPs can be efficiently used for identification, imaging and tracking of pathogens and disease development [234]. Fluorescent NPs can be used for imaging of the early stage of chronic diseases such as cancer. Magnetic NPs can be used in advance detection techniques such as MRI. For instance, biofabricated-AuNPs from *Candida albicans* have been used as a probe for liver cancer cells. The AuNPs specifically bound to cancer cells only and facilitated in differentiating cancer cells from normal cells [235]. Thus, microbe-based NPs have a significant role in disease diagnosis; however, their application in detection and diagnosis is still in a developing stage, and rigorous studies are required for their applications in this field.

### 6.3. Application of Nanoparticles in Food Industry

NPs can be used in the food industry in two important areas, food packaging and food processing [236,237]. For instance, Espitiaet et al., 2012 blended zinc oxide NPs with polymeric material for the synthesis of packaging material. The newly synthesized packaging tissue showed improved antibacterial characteristics [237]. Similarly, other studies also suggested that zinc oxide NPs could be employed for synthesizing containers and nutritional covers for packaging food materials [238,239]. The use of nanomaterials in packaging materials prevents contamination and keeps the food item fresh [2]. AgNPs have a specific capability to pierce the biofilm of bacterial cells, which provides them with resistance against stress. Hence, AgNPs can also be deployed in the decontamination and cleaning process during food packaging [240].

### 6.4. Applications of Nanoparticles in Agriculture

NPs have applications in agriculture as nanofertilizers, nanopesticides and nanoinsecticides [2]. Some of the important applications of NPs in the agriculture sector are discussed below.

#### 6.4.1. Nanoparticles as Fungicides

NPs of metal and metal oxides have excellent antifungal properties. Fungi are the most damaging plant pathogens which cause several diseases in plants. In some recent reports, it has been observed that copper and copper oxide NPs biofabricated from *Streptomyces* spp. showed a good antifungal property against various pathogenic fungi, such as *Pythium ultimum*, *Alternaria alternata*, *Fusarium oxysporum* and *Aspergillus niger* [121,149]. In another study, Kaur et al., 2018 tested the antifungal activity of biosynthesized AgNPs (synthesized from *Pseudomonas* sp. and *Achromobacter* sp.) against *Fusarium oxysporum* infection in chickpea [241]. In this study, biosynthesized AgNPs were applied in in vivo and in vitro pot experiments, and it was observed that AgNPs exhibited very strong antifungal action against *Fusarium oxysporum.* Hence, NPs can be effectively used as fungicides for treating fungal diseases in plants. However, the dose of NPs and their toxicity are still major issues for the commercialization of NPs as fungicides.

#### 6.4.2. Nanoparticles as Fertilizers

The ever-decreasing soil fertility due to overuse of chemical fertilizers is a major issue in the agricultural sector. NPs can be used for the production of alternative nanofertilizers which are nontoxic, efficient and ecofriendly [2]. In a recent report, Bisinoti et al., 2019 used carbon-based nanomaterials as fertilizers in soil and concluded that the nanomaterial-based fertilizers could reduce the use of chemical fertilizers [242]. In other studies, Guo et al., 2018 elucidated that zeolites and nanoclay minerals or crystals can also be used as fertilizers [243]. Subbarao et al., 2013 demonstrated that the coating of nanomaterial over potash fertilizer resulted in its slow release [244], which can reduce the loss of fertilizer and minimize the overuse of chemical fertilizers [245].

#### 6.4.3. Nanoparticles as Pesticides

NPs can also be used for the production of nanopesticides. Nanopesticides may contain NPs in the form of micelles, particles, nanopolymers (organic constituent) and metal oxides (inorganic constituent). In recent years, many new preparations containing NPs have been produced and used as pesticides. It has been explained in many studies that metallic NPs can be used as effective pesticides against various insects and pests, which incur yield losses in agriculture across the globe [246,247,248,249]. For instance, Wang et al., 2007 demonstrated that oil–water (O/W) based novel nanoemulsion could be effectively used as a carrier for the pesticide beta-pypermethrin (beta-PP). This study concluded that beta-PP coated on O/W nanoemulsion spread more efficiently than normal beta-PP and reduced the overall use of this pesticide [250]. In another study, Goswami et al., 2010 observed that the diseases caused by the baculovirus *Bombyx mori* nuclear polyhedrosis virus and *Sitophilus oryzae* in silkworm can be controlled by various NPs, such as zinc oxide, silver, aluminum oxide and titanium oxide NPs [247]. Hence, NPs can be effectively used as pesticides. However, the dose of NPs and their toxicity to plants are still major issues for the commercialization of NPs as pesticides.

#### 6.4.4. Applications of Nanoparticles in Bioremediation

The remediation of highly persistent and xenobiotic water pollutants like cationic dyes, acid dyes, azo dyes and other such pollutants is crucial for wastewater treatment and its future use. These pollutants enhance the water pollution and negatively affect aquatic life. NPs have a larger surface area and smaller size and can therefore either act as catalyst or adsorb the pollutants over their larger surface area. In many reports, the catalytic properties of some NPs coupled with biological components have been evaluated for reducing toxic pollutants [251,252]. Sharma et al., 2015 observed that AgNPs efficiently decolorize organic dyes through catalytic activity. They showed that NPs can be used as a catalyst in industries for the degradation of organic dyes with high efficiency [253]. It has been reported that both Ag and AuNPs exhibit acceptable catalytic activity in the removal of organic dyes. These NPs decrease the time required for the removal of dye and efficiently enhance the rate of reaction [254,255]. Bhargava et al., 2016 demonstrated that AuNPs can also be used as an adsorbent for organic dyes. It was observed that AuNPs having surface proteins synthesized from fungus *Cladosporium oxysporum* AJP03 efficiently improved the adsorption of rhodamine-B organic dye [152]. Recently, Koul and Taak (2018) described the roles of TiO_2_NPs, FeNPs, bimetallic NPs, magnetic NPs, nanoclays, nanotubes and nanosponges in soil bioremediation. The authors emphasized that the green synthesis of NPs could be an effective way to treat soil and water pollution [256].

## 7. Future Perspectives and Conclusions

Microbial cells are fast growing, easy to maintain and can be used for the safer and rapid production of NPs of a desired nature and structure. However, the biosynthesis of NPs via microbial synthesis has many drawbacks, which may include achieving a homogenous shape, symmetry, composition and size of NPs which is governed by environmental conditions, such as temperature and pH of the medium [71]. Although recent studies have shown the huge potential of microbes for the synthesis of novel NPs and their use in biomedicines and cancer treatment [257,258,259], the methods of biosynthesis using microbes need to be modified for commercial production, and the synthesis of NPs needs to be scaled up compared to traditional methods. Moreover, an in-depth investigation is required to study the toxicity, biocompatibility and potential effect of NPs on the immune system, respiratory system, hepatobiliary system, reproductive system, kidney, eyes, skin and other organs [260]. The use of genetically engineered microorganisms for the fabrication of NPs of a desired shape and size is novel in this arena. Genetically engineered organisms have several advantages over the conventional bioproduction of nanomaterials, such as the rate of biosynthesis, cost of production and energy efficiency. Although the biofabrication of NPs using genetically engineered organisms has many advantages, such as environmental safety, cost effectiveness and feasibility of NP production, the use of recombinant organisms is still very scarce. Issues like reproducibility, purity and separation of NPs and the survival of recombinant strains are still required to be addressed to improve NP synthesis using genetically engineered organisms. Moreover, researchers also face challenges related to public acceptance, biosafety, biosecurity and transgene escape when using genetically engineered organisms for the fabrication of NPs [75]. However, it is expected that in the near future, the biofabrication of NPs using genetically engineered organisms will receive significant attention and will be a method of choice for the sustainable production of nanomaterials. Thus, NPs have immense potential in different applications. However, factors such as toxicity, doses and host immune response during treatment are still required to be addressed prior to the commercialization of NPs.
